# Effect of Immunoglobulin Therapy on the Rate of Infections in Multiple Myeloma Patients Undergoing Autologous Stem Cell Transplantation or Treated with Immunomodulatory Agents.

**DOI:** 10.4084/MJHID.2010.005

**Published:** 2010-04-20

**Authors:** Alhossain Khalafallah, Matthias Maiwald, Amanda Cox, Denise Burns, Gerald Bates, Terry Hannan, David Seaton, Bernadene Fernandopulle, Damien Meagher, Terry Brain

**Affiliations:** 1 Launceston General Hospital, Launceston, Tasmania; 2 School of Human Life Sciences, University of Tasmania, Australia; 3 Department of Pathology and Laboratory Medicine, KK Women’s and Children’s Hospital, Singapore; 4 University of New South Wales, Australia; 5 Mersey Hospital, Latrobe, Tasmania, Australia

## Abstract

Multiple myeloma (MM) is associated with a significant risk of infection due to immune dysfunction. Infections are a major cause of morbidity and mortality in MM patients. There are few data available regarding the prevalence of infection in MM patients, especially in conjunction with newer generations of immunomodulatory drugs (thalidomide, bortezomib, lenalidomide) or post autologous stem cell transplantation (ASCT). Intravenous immunoglobulin (IVIG) has been used successfully to reduce infection rates in the stable phase of MM, with limited data in other stages.

We retrospectively analyzed 47 patients with MM from March 2006 to June 2009 at our institution. All patients received thalidomide and steroid therapy for at least 6 months. Nine patients received bortezomib and 11 lenalidomide subsequent to thalidomide, because of disease progression, and 22 patients underwent ASCT. The median age was 64 years (range 37–86), with a female–to-male ratio of 18:29. The median residual-serum IgG-level at time of infection was 3.2 g/L, IgA 0.3 g/L and IgM 0.2 g/L. Most patients suffered from recurrent moderate to severe bacterial infections, including the ASCT group. Fifteen patients suffered from different degrees of viral infections.

All patients except 3 received IVIG therapy with a significant decline of the rate of infection thereafter (p<001). Our analysis shows that patients with MM treated with the new immunomodulatory drugs in conjunction with steroids are at significant increased risk of infection. Employing IVIG therapy appears to be an effective strategy to prevent infection in this cohort of patients. Further studies to confirm these findings are warranted.

## Introduction:

Multiple myeloma (MM) is a malignant tumor of plasma cells associated with impaired function of immunoglobulins, which are an essential component of the immune system.^1^,^2^ Patients with MM are at increased risk of infection, due to a combination of several factors, including immunoparesis and physical factors.^2^,^3^

MM and chronic lymphocytic leukemia patients have been shown to have a 15-fold increased rate of infection compared to age-matched patients.^4^,^5^ Infection is a major cause of death in MM patients, with 18–33% of serious infections resulting in death.^4^,^5^ The types of infections that affect MM patients are predominantly bacterial, affecting the respiratory and urinary tracts as well as causing septicemia.^1^–^5^ Furthermore, patients with MM undergoing stem cell transplantation are at additional risk of viral reactivation as well as opportunistic bacterial infections.^5^,^6^

MM patients with hypogammaglobulinemia and those who have been actively treated with chemotherapy are at high risk of bacterial infection.^7^,^8^ Furthermore, MM patients are also susceptible to systemic and localized viral infections.^9^ Due to the high rate of disease relapse, many serious infections present after the initial period of chemotherapy.^8^.^10^ There are some data suggesting that the use of intravenous immunoglobulins (IVIG) during the stable phase of MM disease can reduce infection rates and improve outcomes;^11^ however, there is a lack of data regarding their use in other disease stages.

IVIG therapy is employed mainly in the management of immunocompromised patients to avoid recurrent infections.^11^–^13^

To date, there is only limited availability of data regarding the effect of ASCT in conjunction with new immunomodulatory drugs (thalidomide, lenalidomide, bortezomib) on the rate of infection in MM patients. This study provides a retrospective analysis of the infection risk and outcomes for patients with MM who underwent ASCT and/or received these new drugs for immunomodulatory therapy. Furthermore, we aimed to evaluate the effect of IVIG therapy on the rate of infection in this cohort of patients.

## Patients and Methods:

This study was conducted at the Launceston General Hospital, a tertiary referral centre for Northern Tasmania, Australia. All patients who presented for treatment of MM during the period between March 2006 and June 2009 were included in the study. The Statewide Human Ethics Committee of Tasmania approved this study. The World Health Organization criteria were used for the diagnosis of MM.^14^ All patients had advanced disease and were undergoing active treatment for MM.

There were 47 patients with a median age of 64 years (range 37–86) and a female to male ratio of 29:18 (**Table 1**). There were 19 patients below 60 years of age and 28 patients above 60 years old. Most of the patients had IgG MM (34 patients), while 4 patients had IgA myeloma, and the remainder (9 patients) had Bence-Jones myeloma. Elevated serum free light chains were present in 25 cases. Serum free light chains were measured using FREELITE Human Kappa and Lambda Free kits (The Binding Site, San Diego, CA) on a Dade Behring nephelometer. The paraprotein levels at diagnosis were assessed, as well as the levels of residual IgG, IgA and IgM. Immunoparesis was defined as non-paraprotein immunoglobulin concentrations below the laboratory lower limit of normal (IgG <7.5 g/L, IgA <0.8 g/L, IgM < 0.5 g/L). There were 22 patients overall who underwent autologous stem cell transplantation (ASCT); 19 as upfront treatment and 3 as salvage therapy for relapsed disease.

The rate of recurrence and severity of infection was recorded for all patients. An acute upper respiratory tract infection (URTI) was defined as the recent onset of rhinorrhea, nasal or sinus congestion, otitis media, pharyngitis and/or cough with clear chest radiograph, and a lower respiratory tract infection (LRTI) included patients with signs and/or symptoms of pneumonia and a new radiographic infiltrate (**Table 2**).

Each infection was classified as bacterial, viral, fungal or unknown, according to clinical, radiological and laboratory findings. Infections were further divided according to severity: severe infections defined as serious infections requiring hospitalization and intravenous antibiotics/antifungals and/or antivirals, or when life-threatening complications occurred including septicemia, pneumonia, or ICU admission; moderate infections were secondary bacterial infections of the upper or lower respiratory tract, urinary tract, skin abscesses, cellulitis or localized herpes zoster that may have also required hospitalization and intravenous antibiotics/antivirals; minor/mild infections were those requiring only oral antimicrobials without hospitalization. Microbiological tests were performed at the Department of Microbiology, Launceston General Hospital.

IVIG is derived from the plasma of blood donors and consists mainly of pooled immunoglobulin G (IgG) with a small amount of immunoglobulin A (IgA) in a maltose solution (Commonwealth Serum Laboratories, Australia).

Of the 47 patients with MM in this study, 44 patients received monthly infusions of IVIG at 0.25–0.4 g/kg body weight (median 24 g; range 12–48 g). Three patients with mild or minor infections and normal IgG levels did not receive any infusions. The patients received monthly IVIG as outpatients and tolerated the therapy well without side effects.

## Results:

The 47 patients included in this study were first diagnosed with MM between the years 2000 and 2008. At the time of diagnosis, the median paraprotein level was 32 g/L (range 15–102), excluding patients with Bence-Jones myeloma who did not have a measureable paraprotein level. The median residual IgG level at time of infection was 3.2 g/L (range 0.3–11.0), median IgA 0.3 g/L (range 0.1–4, excluding patients with IgA myeloma), and median IgM 0.2 g/L (range 0.1–1.1). Each median is below the normal reference range as determined by our laboratory, which is 7.5–15.6 g/L for IgG, 0.8–4.5 g/L for IgA and 0.5–2.6 g/L for IgM. The median neutrophil count at the time of infection was measured at 3.4/nL (range 1.5–15.9), with the normal neutrophil range being 2.0–7.5/nL of the 47 patients, 17 patients were positive for Bence-Jones proteinuria, including the 9 patients with Bence-Jones myeloma. Throughout the course of their disease, 25 patients had elevated serum free light chains with a mean peak level of free kappa of 523 mg/L and free lambda of 697 mg/L. All 47 patients were actively treated with thalidomide for at least 6 months, and all received different doses of steroids during their course of treatment, when the infections occurred. Subsequently, nine patients received bortezomib and 11 received lenalidomide for relapsed or progressive disease. Thirty-three patients were at high risk of developing *Pneumocystis jirovecii* pneumonia due to prolonged treatment with intermediate- to high-dose steroids (40 mg oral dexamethasone for 4 consecutive days, 3 times monthly) and therefore received standardized trimethoprim and sulfamethoxazole prophylaxis. Prophylactic antiviral treatment with famcyclovir was started for 23 patients with a history of herpes simplex and/or varicella zoster virus (VZV) reactivation. Prior to IVIG therapy, 4 patients developed generalized VZV infection and received IV acyclovir for 10 days followed by maintenance famciclovir orally. Localized recurrent herpes zoster infection was noted in 11 patients and required ongoing antiviral treatment. After IVIG therapy, there were no recorded admissions for patients with either generalized or localized VZV infections. However, these patients were commenced on concomitant antiviral prophylaxis after the occurrence of these infections.

Prior to the introduction of IVIG therapy, most of the patients were admitted to hospital for a period extending from a few days to several weeks with various types of infection that required intravenous antibiotic or antiviral therapy. The median length of stay was 12 days (range 1–35 days). Five patients presented with mild infection that required oral antibiotics only. There were 26 patients assessed as having mild to moderate infection that required antibiotics, while 8 patients experienced moderate to severe infection that required hospitalization and intravenous antibiotics and antifungals, 3 of these required additional ICU support. Furthermore, the patients who developed severe infection requiring ICU admission had undergone ASCT and were in the first 12 months post transplant. Most of the patients suffered from URTI (40 patients) and/or LRTI (36 patients), while 8 patients complained of recurrent urinary tract infections (UTI). Most of the cases of mild to moderate infections required oral antibiotics, with complete resolution of the infection thereafter. There were 3 cases diagnosed with septicemia; the organisms were *Staphylococcus* species (2 patients) and *Enterococcus* species (1 patient). Central intravenous lines were inserted after the occurrence of infections in 6 patients who had poor venous access, in order to facilitate the regular use of IVIG therapy. These lines have not been associated with any recorded case of septicemia to date, based on blood cultures withdrawn from these catheters. All patients who underwent ASCT experienced mild to severe bacterial infections and suffered at some stage from LRTI prior to IVIG therapy. Furthermore, 10 patients in the ASCT group developed localized (6 patients) or generalized (4 patients) herpes zoster infections prior to IVIG therapy at a variable time of onset after transplant, with a median onset of 16 months (range 6–20). Median onset of all infections (bacterial and viral) in the ASCT group was 12 months post-transplant (range 2–20). Following IVIG therapy, the median residual IgG level among the patients significantly improved to 5.5 g/L (range 1.2–16.2) (p=0.03), while the median paraprotein level after myeloma treatment decreased to 8 g/L (0–47).

We observed that MM patients who received IVIG therapy did not have recorded hospital admissions for severe infection after commencement of the IVIG, with a median observed interval post commencement of IVIG therapy of 10 months (range 6–33). However, mild-moderate infections were observed in 16 patients who developed URTI (10) and LRTI (6) that required intravenous antibiotics and hospitalization with successful control of their infections. The risk of bacterial and viral infection was greatly lowered after IVIG therapy, with a risk ratio of 0.13 (95% CI 0.09 to 0.19; p<0.001) estimated by Poisson regression.

Among the patients who received IVIG therapy, 33 were in remission, 2 had stable disease and 12 had progressive disease. Most of the patients (43) received concomitant monthly intravenous bisphosphonate therapy during administration of IVIG. All patients who received IVIG were actively treated for myeloma disease with either one of the immunomodulating agents (thalidomide, bortezomib, lenalidomide) at the time of infection. There were 24 patients in consolidation on therapy with thalidomide, while 20 patienst were treated for disease progression with bortezomib and lenalidomide and three patients were treated by induction treatment for myeloma disease with thalidomide and dexamethasone. After a median follow-up of 36 months (range 8–106), 40 patients were alive (86%), 2 of these were in complete remission, 9 in near-complete remission, 22 in partial remission, 2 with stable disease and 5 with progressive disease. Seven patients succumbed to their primary disease; two transformed to plasma cell leukemia and died as a consequence of disease progression, two developed multi-organ failure including respiratory and renal failure that required hospitalization with associated chest infection, while 3 died from cardio-respiratory failure during disease progression at home.

## Discussion:

In the new era of immunomodulatory therapy for MM, employing thalidomide, bortezomib and lenalidomide, there is an additional modification of the immune response of the hosts. There is an increased risk of viral infection in MM patients who are receiving bortezomib.^9^ Other data suggest that thalidomide has an effect on T-cells and is associated with opportunistic infections.^15^ Thalidomide is an antiangiogenic agent that inhibits the production of interleukin (IL)-6 and activates T cells to produce IL-2 which alters the number and function of natural killer cells.^15^,^16^ Furthermore, thalidomide has been shown to have anti-tumor necrosis factor alpha (TNF-α) effects, which increases the risk of opportunistic infection.^15^ Lenalidomide is an immunomodulating drug of the IMiDs class which has a similar mechanism of action to thalidomide. The drug has a different toxicity profile that includes neutropenia, an additional risk factor for infection.^17^–^19^ Lenalidomide also shows anti-inflammatory effects, with downregulation of TNF-α and pro-inflammatory cytokine production.^20^,^21^ A recent safety study of lenalidomide as a single agent in the treatment of MM recorded 60% grade 3–4 neutropenia with only 4% of patients suffering from severe infections.^19^ The cumulative effect of addition of dexamethasone therapy reportedly increases the risk of infection to 11.3% using lenalidomide plus dexamethasone versus 6.2% in the dexamethasone alone treated group.^22^ Bortezomib is a proteasome inhibitor with an inhibitory effect on T cell proliferation.^18^,^23^ The possible effect of bortezomib on cell-mediated immunity is likely responsible for the increased incidence of herpes zoster infections found in MM patients.^9^

In our series, 47 patients with MM were treated with thalidomide in addition to steroids. The steroids were used concomitantly to the immunomodulatory therapy to increase the synergistic effect.^16^–^18^,^22^,^23^ Patients received dexamethasone doses between 20 mg and 40 mg orally 1–2 weekly for at least 6 months (23 patients) or prednisolone 25 mg orally daily or every alternate day (24 patients) for at least a period of 6 months, concomitantly to the immunomodulatory drugs. Prior to IVIG therapy, 42 MM patients, including the ASCT group, suffered from different degrees of mild to moderate infections (55%), and moderate to severe infections (17%) (Table 2). The relatively high incidence of infection rates in our study are mainly due to the facts that we registered all occurrence of mild and moderate infections and also included the ASCT group in the analysis as well as relapsed patients who were receiving different immunomodulatory agents. There was no significant difference in the prevalence of infections in patients treated with thalidomide compared to lenalidomide or bortezomib. It is worth noting that the residual normal IgG levels improved significantly (p=0.03), with further improvement of IgA and IgM levels, after IVIG therapy. However, the low levels of IgG remained the surrogate marker for immunodeficiency and susceptibility for infections. The rate and severity of infections, hospital admission and use of intravenous antibiotics/antivirals were reduced significantly after commencement of monthly IVIG therapy (p<0.001). Our results are largely in concordance with a meta-analysis of 6 different randomised trials studying the effect of IVIG therapy in different patient populations with CLL and MM, which showed no survival benefit, but there was a significant decrease in the occurrence of major and clinically documented infections.^24^ However, the patients populations and therapy received in these studies are different from our series.

## Conclusions:

In summary, our data suggest that IVIG therapy may play an important role in the supportive management during the active treatment of MM patients, especially those receiving new immunomodulatory agents or ASCT. Accordingly, there was a significant benefit of IVIG therapy in MM patients with a corresponding decrease in hospital admission for recurrent infections or use of intravenous antibiotic and or antiviral therapy. Further trials studying the effect of IVIG in this cohort of patients are warranted.

## Figures and Tables

**Table 1:** Patient characteristics.

Total number of patients	47

Sex	
Male	29
Female	18

Age	Median 64 (range 37–86) years
<60	19
>60	28

Disease Type	
IgG	34
IgA	4
Bence Jones myeloma	9
Concomitant positive free light chains	25

Median paraprotein level at diagnosis	32 (range 14.9–102) g/L
Median residual IgG level at time of infection	3.2 (range 0.3–11) g/L
Median IgA level at the time of infection	0.3 (range 0.1–4) g/L
Median IgM Level at the time of infection	0.2 (range 0.1–1.1) g/L
Median neutrophil count at the time of infection	3.4 (range 1.5–15.9)/nL
Median lymphocyte count at the time of infection	1.7 (range 0.3–5.1)/nL

Mean serum free kappa light chain	523 mg/L
Mean serum free lambda light chain	697 mg/L

Treatment received:	
ASCT (upfront)	19
ASCT (relapsed)	3
Thalidomide	47
Bortezomib	9
Lenalidomide	11

Disease status during occurrence of infection	
Consolidation /maintenance therapy	24
Treatment for relapsed disease	20
Induction therapy	3

Total IVIG dose (0.25–0.4g/kg body weight)	24 g (range 12–48)

Patients receiving steroids:	
Dexamethasone	23
Prednisolone	24

IVIG=intravenous immunoglobulin.

**Table 2:** Summary of infections and outcome in patients who were actively treated for multiple myeloma. URTI=upper respiratory tract infection, LRTI=lower respiratory tract infection, GITI=gastro intestinal tract infection; UTI=urinary tract infection, ICU=Intensive Care Unit, IVIG=immunoglobulin therapy.

Types of infection	prior to IVIG therapy	after to IVIG therapy
URTI (recurrent)	40	10
LRTI (recurrent)	36	6
GITI	3	0
Septicemia	3	0
UTI	8	0
Oral *Candida* infection	2	0
Cholecystitis	1	0
Viral infection		
Localized herpes infection	19	2
Generalized varicella zoster infection	4	0

Length of admission for infection	Median 12 days (range 1–35)	Median 3 days (range 1–7)

ICU admission	Three patients required ventilation for severe pneumonia	One patient admitted to ICU for multiorgan failure*

Degree of bacterial infection		
Severe	8/47 (17%)	0*
Moderate	26/47 (55%)	16/47 (34%)
Mild or minor	13/47 (28%)	10/47 (21%)
Concomitant history of viral infection	23/47 (48%)	2/47 (4%)

Rate of infection in the ASCT group		
Moderate-severe bacterial infection	11/22 (50%)	
Mild-moderate bacterial infection	11/22 (50%)	
Viral infection	10/22 (48%)	
Median onset of all infections post transplant	12 months (range 2–20)	

Median residual IgG level after IVIG therapy	5.5 (range 1.2–16.2) g/L	
Median residual IgA level after IVIG therapy	0.4 (range 0.1 -3.7) g/L	
Median residual IgM level after IVIG therapy	0.3 (range 0.1–1.0) g/L	

Median length of follow up for MM disease	36 months (range 8–106)	
Median length of follow up after IVIG	10 months (6–33)	

Outcome of the disease		
Complete remission (CR)	2	
Near CR	9	
Partial remission	22	
Stable disease	2	
Progressive disease	5	
Deceased due to disease progression	7 (2* with concomitant infection)	

* Two patients developed pneumonia when they presented in terminal disease progression with severe pancytopenia (neutrophils <0.5/nl) and multiorgan failure with almost complete bone marrow replacement by plasma cells. These patients were not considered as infection secondary to hypogammaglobulinemia.
